# Adult Learning of Novel Words in a Non-native Language: Consonants, Vowels, and Tones

**DOI:** 10.3389/fpsyg.2018.01211

**Published:** 2018-07-24

**Authors:** Silvana Poltrock, Hui Chen, Celia Kwok, Hintat Cheung, Thierry Nazzi

**Affiliations:** ^1^Université Paris Descartes, Sorbonne Paris Cité, Paris, France; ^2^CNRS, Laboratoire Psychologie de la Perception, Paris, France; ^3^Department Linguistik, Universität Potsdam, Potsdam, Germany; ^4^Department of Linguistics and Modern Language Studies, The Education University of Hong Kong, Tai Po, Hong Kong

**Keywords:** word learning, minimal pairs, non-native speech perception, tones, adults

## Abstract

While words are distinguished primarily by consonants and vowels in many languages, tones are also used in the majority of the world's languages to cue lexical contrasts. However, studies on novel word learning have largely concentrated on consonants and vowels. To shed more light on the use of tonal information in novel word learning and its relationship with the development of phonological categories, the present study explored how adults' ability to learn minimal pair pseudowords in a tone language is modulated by their native phonological knowledge. Twenty-four adult speakers of three languages were tested: Cantonese, Mandarin, and French. Eye-tracking was used to record eye movements of these learners, while they were watching animated cartoons in Cantonese. On each trial, adults had to learn two new label-object associations, while the labels differed minimally by a consonant, a vowel, or a tone. Learning would therefore attest to participants' ability to use phonological information to distinguish the paired words. Results first revealed that adult learners in each language group performed better than chance in all conditions. Moreover, compared to native Cantonese adults, both Mandarin- and French-speaking adults performed worse on all three contrasts. In addition, French adults were worse on tones when compared to Mandarin adults. Lastly, no advantage for consonantal information in native lexical processing was found for Cantonese-speaking adults as predicted by the “division of labor” proposal, thus confirming crosslinguistic differences in consonant/vowel weight between speakers of tonal vs. non-tonal languages. These findings establish rapid novel word learning in a non-native language (long-term learning will have to be further assessed), modulated by native phonological knowledge. The implications of the findings of this adult study for further infant word learning studies are discussed.

## Introduction

**Figure 1:**
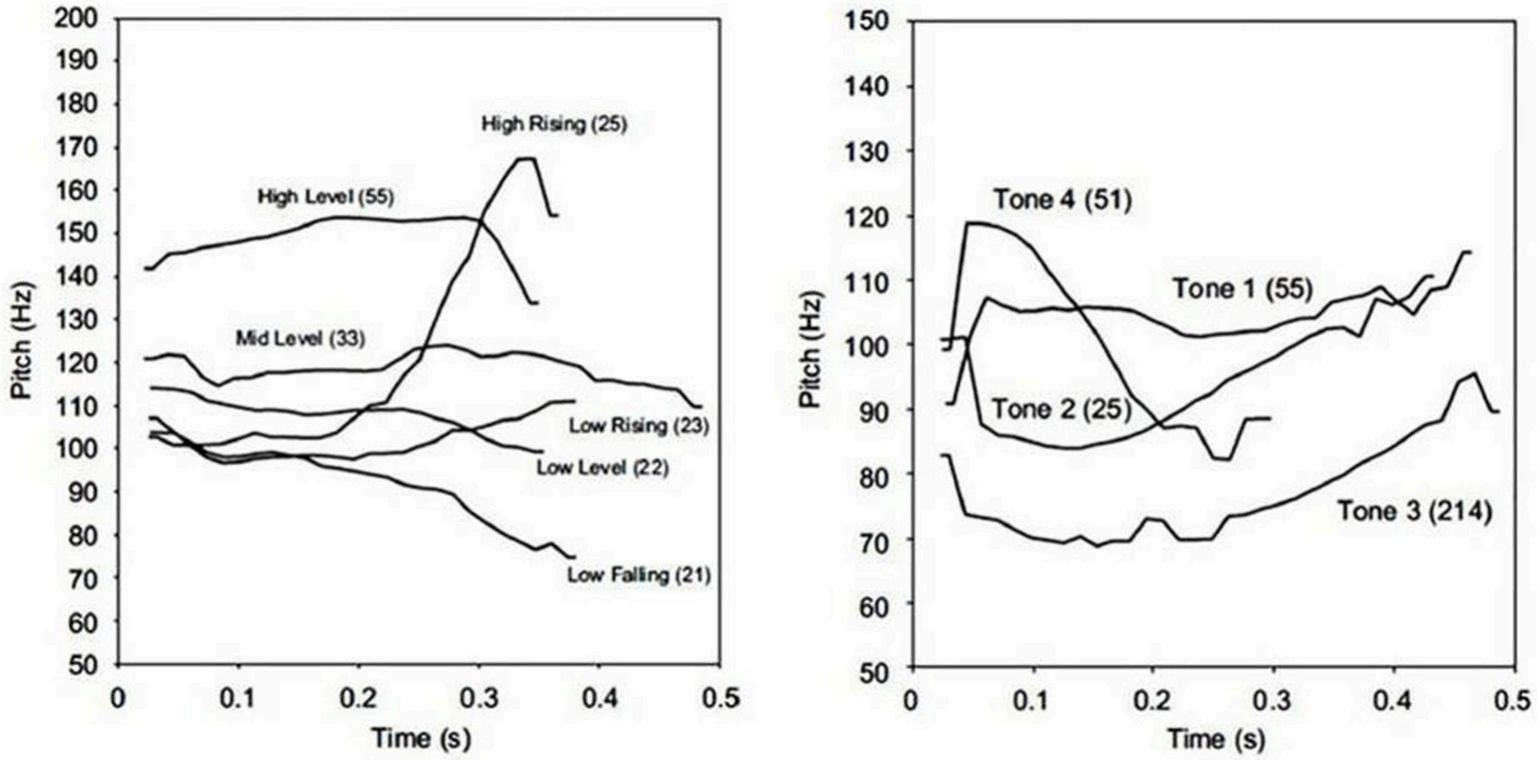
F0 contours for tones in Hong Kong Cantonese (**left**, produced by a male speaker on the syllable 'ji' /ji/) and in Beijing Mandarin (**right**, produced by a male speaker on the syllable'da' /ta/). Note that, because these Cantonese and Mandarin tones come from different speakers, absolute F0 levels cannot be compared across languages, only relative levels are comparable. Images come from a previously published article (Francis et al., 2008), reproduced here with the authors' permission.

Learning words is a crucial step in learning a language, no matter whether it is one's initial, native language as an infant, or a new, non-native language as an adult. Importantly, learning new words requires the ability to process relevant phonetic information and represent it in proper phonological categories. This ability is largely based on which phonetic variations are relevant to word meaning and how phonological categories are established in one's native language. In all languages, lexical representations include segmental information related to the identity of consonants and vowels constituting the word forms. In many languages, lexical representations also include suprasegmental information, such as lexical stress, pitch accents or tones. Phonological repertoires vary across languages, and the same is true for lexically-relevant prosodic information. For instance, both Cantonese and Mandarin have tonal systems, but their systems differ in both number and identity of tones (Wang, 1963; Mandarin: Cheng, 1966; Hashimoto, 1972; Howie, 1976; Cantonese: Bauer and Benedict, 1997; Duanmu, 2000; Yip, 2002), as illustrated in Figure 1. The present study will explore the interplay between word learning and phonological processing (of consonants, vowels and tones) by comparing three groups of adults when learning minimal pairs of Cantonese words in their native (Cantonese-speaking adults) vs. a non-native (Mandarin- and French-speaking adults) language.

Decades of research have established that speech perception becomes language-specific during the first year of life, as attested by decreases in ability to discriminate many (though not all, see below) non-native phonological contrasts (that is, contrasts not used in one's native language), and increases in the ability to discriminate native contrasts. These changes have been found to happen later for consonants (by 10–12 months of age, e.g., Werker and Tees, 1984a; Best et al., 1988; Rivera-Gaxiola et al., 2005), than for vowels (by 6 months of age, Kuhl et al., 1992; Polka and Werker, 1994). The developmental timing for tones is less clear, as changes are usually reported around 10 months of age (Mattock and Burnham, 2006; Mattock et al., 2008; Liu and Kager, 2014; Cabrera et al., 2015) although evidence for changes as early as 4 months has been found in one study (Yeung et al., 2013).

These developmental changes in speech perception, which attest to the early acquisition of the phonological repertoire of the native language, have continued effects in adulthood. Speech perception difficulties have been found for the processing of non-native consonants (e.g., Werker and Tees, 1984b), non-native vowels (e.g., Polka, 1995), and non-native tones (Gandour et al., 2000; Hallé et al., 2004; So and Best, 2010). This is attested by the fact that adults will sometimes have difficulties identifying some non-native sounds, and/or discriminating between non-native sounds. For example, regarding consonant perception, the fact that the English /r/ consonant does not have an equivalent in both Japanese and German has been found to lead to differences in how Japanese- and German-speaking adults perceive this sound (in contrast to English /l/) when compared with English-speaking adults (e.g., Miyawaki et al., 1975; Iverson et al., 2003). Moreover, perception of this non-native contrast differs across the two language groups, with more difficulty observed for the Japanese-speaking adults, who appear to form only one sound category, compared to the German-speaking adults who perceive two sound categories (e.g., Iverson et al., 2003). This further shows that these processing difficulties stem from interference with the native phonological system. With respect to tones, many studies have found that speakers of languages that do not use tone contrasts at the lexical level identify and discriminate non-native tones with more difficulty than speakers of tonal languages (Gandour et al., 2000; Hallé et al., 2004; So and Best, 2010). Even though some discrimination ability is found in speakers of non-tonal languages, they appear to process tones differently. This is attested, for example, by the fact that (non-tonal) French-speaking adults, while being able to discriminate non-native Mandarin tones, perceive these tones less categorically than Mandarin-speaking adults. Some have proposed to link this to their lack of phonological categories for tones (Hallé et al., 2004).

The first goal of the present study was thus to explore the effects of such perceptual changes on adults' ability to learn new words in an unfamiliar language. Although this is a situation that adults have to cope with when learning a new language, it has received surprisingly little attention to this day (but see Chandrasekaran et al., 2010; Cooper and Wang, 2012, 2013, for training studies on English-speaking adults' processing of Cantonese or Mandarin tones in lexical contexts). Here, we evaluated monolingually-raised Mandarin- and French-speaking adults' ability to learn new words in Cantonese, and compared their performance to baseline data from Cantonese-speaking adults. This was done in a laboratory setting, during which, on each trial, adults had to learn a pair of Cantonese pseudowords that differed by either a consonant, a vowel or a tone. Given the above language specialization findings at the perceptual level, evidenced by difficulties in low-level (discrimination or identification) non-native processing, we predict that adults (and infants from 6 to 12 months onward) should have more difficulty learning new words in a non-native language than in their native language, because they are made up of some sounds that do not belong to the native phonological repertoire. Hence, overall performance should be higher for Cantonese-speakers than for Mandarin- and French-speaking adults, and it might even be that the latter two groups fail at learning. Another possibility is that linguistic distance between the native language and the language of the stimuli affects performance. Since Cantonese and Mandarin, being both Sino-Tibetan languages, share many phonological, morphological, and syntactic properties (Li, 1937; Gong, 1980; DeLancey, 2009), which is not the case with Cantonese and Indo-European French, Mandarin-speaking adults might have higher overall performance than French-speaking adults.

One important feature of our experimental design is the fact that on each trial adults had to learn a pair of new pseudowords. Therefore, for learning to take place, adults had to process the phonological contrast distinguishing the two paired words, which allowed us to explore in more detail the interplay between phonological and lexical processing in this process of acquiring new words. To begin with, the fact that the pseudowords contrasted in either consonant, vowel, or tone information allowed us to evaluate differential processing of these three phonological sound categories. This second goal of the present study was motivated by the proposal that consonants carry more information about the lexicon, whereas vowels play a more important role in syntactic and prosodic processing (Nespor et al., 2003). For example, in word reconstruction studies in which English, Dutch, and Spanish listeners hear pseudowords and have to transform them into real words, they preserve consonantal over vocalic information, changing kebra into cobra rather than zebra (van Ooijen, 1996; Cutler et al., 2000). Evidence for this bias for consonantal information in lexical processing (often referred to as the C-bias in the literature) is supported by studies with adults across several non-tonal languages (Dutch, English, French, Italian, Spanish) and a variety of different methods such as word learning (e.g., Bonatti et al., 2005; Creel et al., 2006; Toro et al., 2008; Havy et al., 2014) and lexical access (e.g., New et al., 2008; Carreiras et al., 2009; Delle Luche et al., 2014; New and Nazzi, 2014).

Importantly though, when this project was started, little was known about the C-bias in non-European languages, and in particular in tone languages. Tone languages provide a particularly interesting test of the C-bias as lexical tones are mostly carried by vowels. This might affect performance, in two opposing ways. Indeed, the need for speakers of tone languages to attend to tones to identify words might increase their attention to the vowels (which carry them), and thus increase the weight given to vowels compared to consonants in tone languages compared to non-tone languages. This might result in a lack of bias or in a reversed advantage in processing vocalic information. In contrast, the fact that vowels carry tones might make the acoustic realization of vowels more variable in tone than in non-tone languages, making them more difficult to process and identify. If so, the consonant bias in lexical processing found in non-tone languages might be even more pronounced in tone languages.

To date, only two studies have explored this issue, but have focused on levels other than word learning: lexical access to known words, and word form segmentation in an artificial language. First, in a word reconstruction study based on van Ooijen (1996) and testing lexical access, Wiener and Turnbull (2016) asked participants to transform a pseudoword into a real word by changing either a consonant, a vowel (in fact, to conform to Chinese phonology, they were asked to change the final - in Chinese phonology, and in the stimuli used in that study, the final corresponds to V, VV, or VVN), a tone, or any of the three. Results show effects of condition, corresponding to the fact that Mandarin-speaking adults appear to preferentially change tones over both consonants and vowels/finals, with vowels appearing to be the less mutable sound category, contrary to what had been found in Dutch, English, and Spanish (van Ooijen, 1996; Cutler et al., 2000). These findings suggest a different balance in the weight given to consonants and vowels, with less weight given to consonants (or more weight given to vowels), by Mandarin-speaking adults. Second, an artificial language study exploring whether Cantonese-speaking adults use consonants or vowels (and tones) to segment a fluent speech stream revealed that they could not use consonantal information alone, but could rely either on vocalic information alone (although the difference between the consonant and vowel conditions was not significant), or more likely on a combination of vocalic and tonal information (Gómez et al., 2017). This also suggests a different balance in the weight given to consonants and vowels by Cantonese-speaking adults as compared to French- or Italian-speaking adults (Bonatti et al., 2005; Toro et al., 2008). The present study will add to this literature by providing the first evaluation of this issue in a word learning task for tonal language speakers, for either the native language (Cantonese adults processing Cantonese stimuli) or a foreign language (Mandarin adults processing Cantonese stimuli). It will also provide the first evidence of whether the C-bias, found in French-speaking adults when processing native stimuli, would also extend to the processing of non-native stimuli (French adults processing Cantonese stimuli).

The results regarding the use of tonal information by French-speaking adults when learning words will also inform our understanding of the link between phonological and lexical processing. Previous studies on tone perception/identification have established that even though adult speakers of non-tonal languages have more difficulties at tone processing than speakers of tone languages (e.g., Gandour et al., 2000; Hallé et al., 2004; So and Best, 2010), they tend to perform above chance levels. Recently, Liu and Kager (2014) found that tone discrimination in non-tonal Dutch-learning infants follows a U-shaped function: while 5–6-month-olds can discriminate a Mandarin tone easily, their sensitivity declines between 8 and 15 months, but they regain sensitivity to it by 17–18 months. They suggested that this rebound in sensitivity might be related to the acquisition of the intonation of the native language. If this increased sensitivity is not limited to low levels of processing, then the French speakers in our experiment might perform at above chance levels on the tone-contrasted trials. This prediction is supported by previous findings on English-speaking adults (Chandrasekaran et al., 2010; Cooper and Wang, 2012, 2013) showing tone processing in lexical contexts, although in these studies, participants were subject to intense word training (several training sessions of about 30 min, in which some feedback was provided). It is unclear whether non-tone language speaking adults would show sensitivity to tone information in a less intensive task.

The present study used eyetracking to investigate the ability of Cantonese-, Mandarin-, and French-speaking adults to learn pairs of pseudowords in Cantonese, while processing fine phonetic information (consonant vs. vowel vs. tone information), whether used contrastively in the native language or not. On each of 24 trials, adults saw a pair of cartoons. In each cartoon, an unfamiliar object was presented visually while 6 sentences in Cantonese, each containing a pseudoword labeling that object, were heard. Between the two cartoons, the pseudowords differed by either a consonant (8 times), a vowel (8 times) or a tone (8 times). Adults were then tested on whether they had been able to learn the words following this short word learning phase, by presenting them with the two unfamiliar objects side-by-side, and observing their pattern of object looking before (prenaming phase) and after (postnaming phase) one of the objects was named. The current procedure was based on Experiment 1a of Havy et al. (2014) in which French-speaking adults were taught pairs of new pseudowords that differed either by a consonant or by a vowel. A comparison of performance in the two conditions to evaluate the consonant bias revealed that adults increased their looking times toward the target object (mean percentage of looking times at the target object in the postnaming—prenaming phase) similarly in both the consonant and vowel conditions, but that latencies in shift from the distractor to the target at the time of naming were faster in the consonant than in the vowel condition, establishing a consonant bias. To explore the relative strength of the processing of consonant, vowel, and tone information in our three linguistic groups, we similarly analyzed changes in mean percentage of looking times at the target object between the prenaming and postnaming phases (which also evaluates whether the pseudowords were learned), and latencies to shift from the distractor to the target at the time of naming. We also performed cluster-based permutation analyses on the time course of looking times in the different conditions to determine when in processing looking times to the target differ among the three conditions.

## Materials and methods

### Participants

Seventy-two adults were tested in total: 24 Mandarin- (age range 21-27 years, mean age: 23.9 years, 21 female), 24 French-speaking adults (age range 21–38 years, mean age: 25.2 years, 12 female), and 24 Cantonese-speaking adults (age range 20–43 years, mean age: 27.4 years, 20 females) who served as the native speaker control group. French- and Mandarin-speaking participants had no knowledge of Cantonese (and French adults had no knowledge of other tone languages either). All participants had grown up monolingual, and no further background information (such as musical abilities…) were collected. French-speaking adults were tested in Paris, Cantonese- and Mandarin-speaking adults were tested in Hong Kong, the latter group within a week of their arrival in Hong Kong. Before the experiment started, informed written consents were obtained from all participants. Both the experimental protocol and consent procedures were reviewed and approved by the CERES (Comité d'évaluation éthique des projets de recherche) of the Université Paris Descartes and the Human Research Ethics Committee of the Education University of Hong Kong. All data were obtained according to the principles expressed in the Declaration of Helsinki.

### Stimuli

#### Speech stimuli

The speech stimuli (presented in Table 1) consisted of 24 pairs of disyllabic CVCV Cantonese pseudowords, differing by a minimal phonological contrast of 1 feature (except for two 2-feature contrasts for consonants, and two 2-feature contrasts for vowels, which could not be avoided due to phonological and lexical constraints in Cantonese). All contrasts were on the first syllable of the words, and the second syllable was always associated with the high level tone (T1). Eight pairs involved a consonant contrast (e.g., **k**^h^ɔ2.lϵ1/−/**t**^h^ɔ2.lϵ1/), 8 involved a vowel contrast (e.g., /p^h^**u**2.fɔ1/−/p^h^**y**2.fɔ1/), and 8 involved a tone contrast (e.g., /p^h^a**5**.mi1/−/*p*^h^*a***6**.mi1/). The tones of the target syllables in the consonant and vowel pairs varied across trials.

**Table 1:** Minimal pairs of pseudowords.

**Condition**		**Pair**	**Feature/Tone change**
Consonant contrasts	1	/tu3.la1/ - /nu3.la1/	Manner, voicing
	2	/mɔ4.hœ1/−/pɔ4.hœ1/	Manner, voicing
	3	/si3.k^h^ɔ1/−/ts^h^i3.k^h^ɔ1/	Manner
	4	/k^h^ɔ2.lϵ1/−/t^h^ɔ2.lϵ1/	Place
	5	/sa1.kɔ1/−/fa1.kɔ1/	Place
	6	/k^h^i1.ka1/−/kw^h^i1.ka1/	Place
	7	/tsu2.mϵ1/−/ts^h^u2.mϵ1/	Aspiration
	8	/k^h^ϵ4.t^h^œ1/−/kϵ4.t^h^œ1/	Aspiration
Vowel contrasts	1	/k^h^iu4.wɔ1/−/k^h^ui4.wɔ1/	Place, roundedness
	2	/sɐ3.kϵ1/−/sɐu3.kϵ1/	Place, roundedness
	3	/fu1.ti1/ - /fɔ1.ti1/	Height
	4	/hœ2.t^h^i1/−/hO2.t^h^i1/	Place
	5	/p^h^u2.fɔ1/−/p^h^y2.fɔ1/	Place
	6	/li3.ts^h^a1/−/ly3.ts^h^a1/	Roundedness
	7	/kϵ1.tsϵ1/−/kœ1.tsϵ1/	Roundedness
	8	/mɐ4.t^h^u1/−/mau4.t^h^u1/	Length
Tone contrasts	1	/mɔ2.li1/−/mɔ1.li1/	T1–T2
	2	/ki2.pa1 - /ki6.pa1/	T2–T6
	3	/pu1.fa1/ - /pu3.fa1/	T1–T3
	4	/ly1.khi1/ - /ly3.khi1/	T1–T3
	5	/tshu1.kϵ1/−/tshu4.kϵ1/	T1–T4
	6	/pha5.mi1/ - /pha6.mi1/	T5–T6
	7	/fœ3.tɔ1/−/f4.tɔ1/	T3–T4
	8	/tϵ4.sɔ1/−/tϵ6.sɔ1/	T4–T6

*T1 (High Level 55), T2 (High Rising 25), T3 (Mid-Level 33), T4 (Low Falling 21), T5 (Low Rising 23), T6 (Low Level 22)*.

While these pseudowords were all contrastive in Cantonese, some were not necessarily contrastive in French and/or Mandarin, as they were likely to assimilate to the same category in those languages, either equally well (such as /**k**^h^ϵ4.t^h^œ1/ - /**k**ϵ4.t^h^œ1/ in French, where [k^h^] and [k] are both allophones of /k/), or with one of the sounds assimilating better than the other (such as /kϵ1.tsϵ1/ - /k**œ**1.tsϵ1/ in Mandarin, which has a front-mid-unrounded vowel [ϵ] but not a front-mid-rounded vowel [œ]). See detailed explanations in the Appendix.

The words were presented in sentences in Cantonese. For the familiarization, they were embedded in a little passage, and appeared in six different sentences. In the test phase, one of the two words was designated twice, in two sentences (see details in “animated cartoons” section below). All speech stimuli were recorded in a quiet room by a female native adult speaker of Hong Kong Cantonese. One audio file of each condition can be find in the Supplementary Material (Consonant trial: /**k**^h^ɔ2.lϵ1/−/**t**^h^ɔ2.lϵ1/; Vowel trial: /p^h^**u**2.fɔ1/−/p^h^**y**2.fɔ1/; Tone trial: /p^h^a**5**.mi1/-/p^h^a**6**.mi1/). Note that while Mandarin and French adults did not speak Cantonese, the structure of the cartoon (with the moving object and the 6 sentences all embedding the target word) made it clear that each target word (which was thus the most frequent content word in each passage) was meant to name the object presented at the same time (which is confirmed by the results, see below).

#### Object stimuli

**Figure 2:**
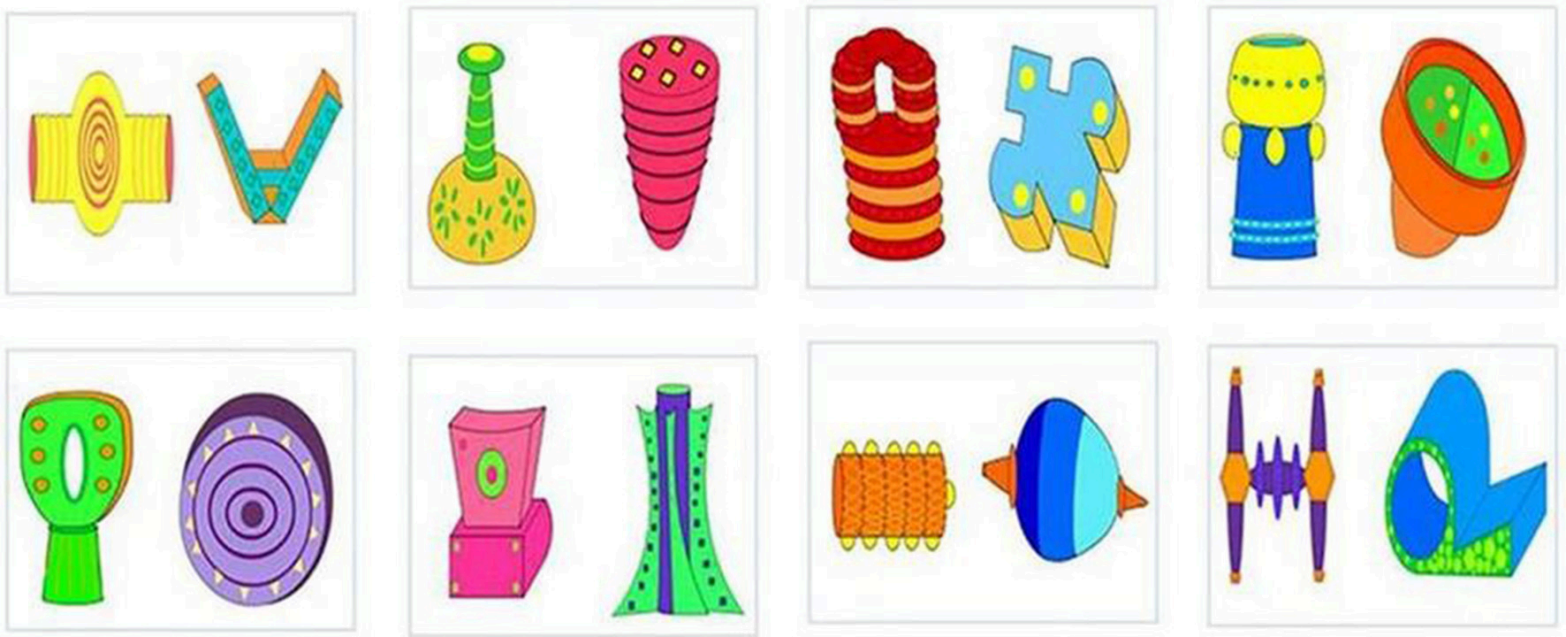
The 8 pairs of novel objects used for word learning.

Images of eight pairs of objects differing in shape, color and texture (see Figure 2) were taken from a previous study by Gonzalez-Gomez et al. (2013). The reason for using clearly different objects was to facilitate learning of the word-object pairings. All objects were selected so that they would look novel to the participants. All 8 object pairs were used 3 times, once in each condition (consonant, vowel, tone). This was done in order to ensure that overall performance differences across conditions could not be due to the objects used.

#### Animated cartoons

**Figure 3:**
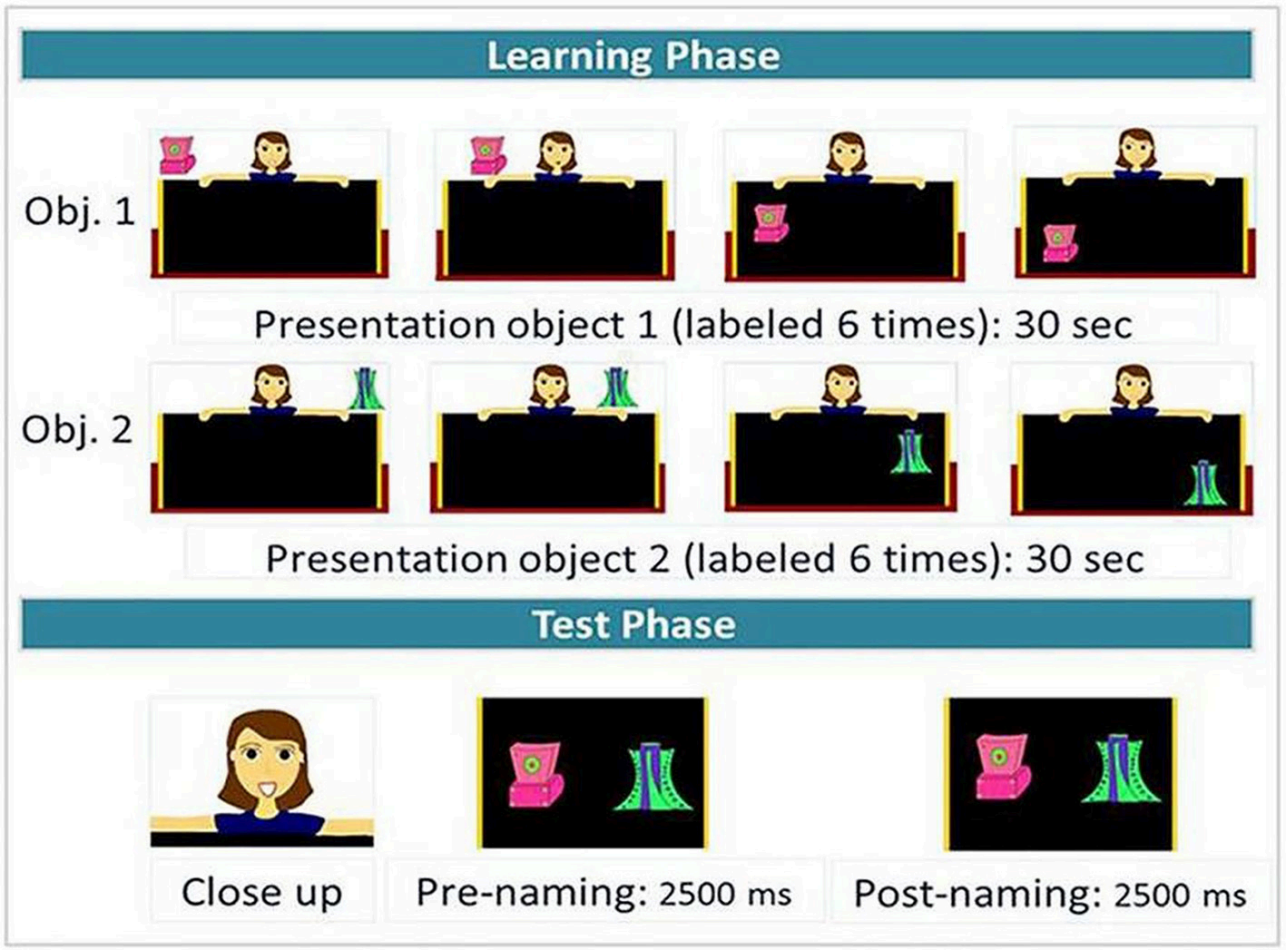
Structure of a word-learning cartoon.

The audio recordings were included in animated cartoons that have been successfully used in a computer-controlled word-learning task in toddlers by Gonzalez-Gomez et al. (2013). An example of a cartoon is illustrated in Figure 3.

On each trial, a female character behind a black board presented the two objects, one at a time (Figure 3, learning phase). The first object always appeared in the left upper corner of the screen. At the beginning, the object moved horizontally in the upper left part of the display, while it was labeled three times (“Look! A [label]! This is a [label]. Look at what I'm doing with the [label]!”). Then, the object started shifting down, while it was labeled one more time (“I'm putting the [label] here”). It started moving vertically in the lower left part of the screen and was labeled two more times (“Have you seen the [label]? Have a look at the [label]!”) before disappearing. The second object was always introduced in the upper right corner of the display and followed a trajectory analogous to that of the first object. The cartoon experimenter followed the objects' movements with her eyes. Participants were successively trained on each label-object pairing for 30 s. The entire learning phase lasted 1 min and each label was repeated 6 times.

After the learning phase, participants were tested immediately on the given contrast. There was a close up on the face of the cartoon experimenter saying: “Look!” in order to direct the participants' fixations to the center of the screen. After the face disappeared, the two objects appeared at the same time, each on the side where it had appeared during the learning phase, and started moving synchronously in a vertical way, for 5000 ms, while the out-of-sight speaker said: “Look at the [target]! Where's the [target]?” about half way through the presentation in order to divide the test phase into a prenaming and a postnaming phase of equal duration (Figure 3, test phase). Since the material was originally designed to test and compare performance in both adults and toddlers, and since it has been shown that it takes 367 ms for infants and toddlers to program eye movements (e.g., Swingley and Aslin, 2000), the cartoons were constructed so that the onset of the *postnaming phase* corresponded to the onset of the first target word + 367 ms for consonant and tone trials; while it corresponded to the onset of the first vowel of the target word + 367 ms for vowel trials (hence, it corresponded to the onset of the contrasting phoneme in all trials). However, for the adult data analyses, we changed the timing by time-locking the onset of the postnaming phase 200 ms after the onset of the contrasting phoneme (as usually done in adult studies, e.g., Barr, 2008), and reducing the size of the *pre-* and *post-naming* phases to 2,000 ms around this time point. Note that since there is debate whether tonal cues are already present in onset consonants, or whether they mostly become available with vowel onset, we conducted a preliminary analysis (see results section “Time course analysis for Cantonese speakers: onset of tonal information use”) to explore this issue in our data.

Every object pair was associated with one pseudoword pair in each experimental condition (e.g., object A and B were associated with /k^h^ɔ2.lϵ1/and/t^h^ɔ2.lϵ1/ in the consonant condition; with /p^h^u.2fɔ1/−/p^h^y2.fɔ1/ in the vowel condition and /p^h^a5.mi1/−/p^h^a6.mi1/ in the tone condition), for use in 24 different trials. Four versions of each cartoon were created so that in half of the trials, object/label A was the target (and consequently object/label B the distractor in those trials) and, in addition, the target was presented as first object in 50% of the trials and as second object in the other 50%. This yielded a total stimulus set of 96 movies, all having a resolution of 1280 × 930 pixel. Presentation of each of the four versions of each cartoon was counterbalanced across participants.

### Apparatus and procedure

In Paris, the movies were presented on a 17″ TFT monitor (1280 × 1024 pixel resolution) with an integrated Tobii T60 eyetracking system which was run by a Dell computer. The presentation of the stimuli and the storing of the data were performed with the Tobii Studio software. In Hong Kong, a Tobii TX300 was used, which was run by a Dell computer and with videos presented on a Tobii TX300 screen unit with a 1920 × 1080 pixel resolution.

Each participant was tested individually in a quiet, dimly lit laboratory room and watched 24 testing trials in total. As French- and Mandarin-speaking participants had no knowledge of Cantonese they received a warm-up trial in Cantonese, in which the two pseudowords used were phonetically different in every single segment (/ka/ - /su/) and in which subtitles were presented, in order to familiarize them with the task.

There were 12 pseudo-randomized orders, which were each presented to two participants in each of the three language groups. Four sub-blocks of 6 trials were presented. After every sub-block, the participant could take a break for as long as s/he wished. In each sub-block, there were 2 consonant trials (one with target on the left, one with target on the right), 2 vowel trials (left, right), and 2 tone trials (left, right). Consequently, within a subject, half of the time the target word was on the left, half of the time it was on the right. All 3 conditions were presented in the first 3 trials and there were never more than 2 target-left or 2 target-right trials in a row. None of the words was presented twice, but the objects occurred three times during the test. Note that the same object pairs were not presented within the same sub-block, in order to prevent learning interference. After creating order 1 with those constraints, it was mirrored to get order 2 (e.g., order 1: trial 1 - trial 24; order 2: trial 24 - trial 1). For order 3, we shuffled the trials of order 1 so that the ones that occurred in the first half in order 1 appeared in the second half (and the other way around). Order 4 was again a mirror of order 3. Orders 5-8 and 9-12 were exactly like orders 1-4 but the conditions were differently assigned following a Latin square design (e.g., order 1: V_pair1_, T_pair8_, C_trial3_ …; order 5: T_pair1_, C_pair8_, V_trial3_ …; order 9: C_pair1_, V_pair8_, T_trial3_ … Note that the number of each pair here means the specific object pair that was used). As a consequence, between-subjects counterbalancing ensured that each object-word pair was presented and tested on the right and left side equally often and occurred in all 3 conditions at the same serial position. The experiment lasted approximately 30 min.

### Data analysis

The eye-tracking data used for the analysis consisted of the binocular gaze position (X and Y coordinates) at each timestamp, that is, every 16.6 ms for French-speaking adults and every 3.3 ms for Mandarin- and Cantonese-speaking adults. Trials in which no data was available for the postnaming phase were discarded from the analyses (27/1728 trials). The data was analyzed in R (version 3.4.3, R Core Team, 2017, http://www.r-project.org) using the *eyetrackingR* package (Dink and Ferguson, 2015, http://www.eyetrackingr.com) for the latency and the growth curve analysis as well as for the cluster-based permutation analysis.

## Results

### Time course analysis for cantonese speakers: onset of tonal information use

**Figure 4:**
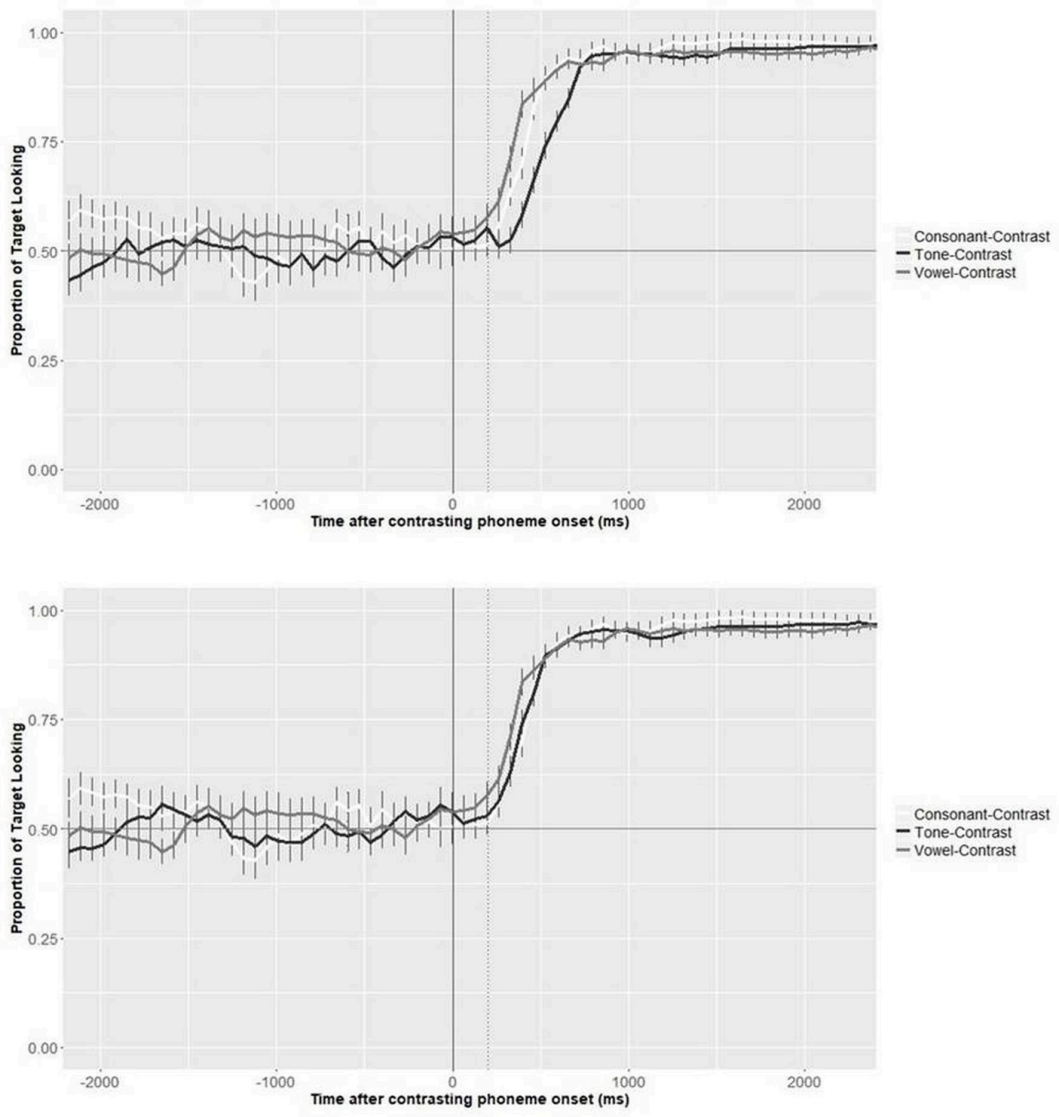
Target looking behavior during the test phase, for Cantonese speakers only. Time point 0 refers to the onset of the first consonant for consonant trials, and the onset of the first vowel for vowel trials in both panels. For tones, time point 0 refers to the onset of the first consonant in the top panel, but the onset of the first vowel for the bottom panel. The dotted line represents the beginning of the postnaming phase.

To evaluate the issue of whether the onset of tones should be time-locked to the onset of the consonants or the vowels of the syllables in which they were embedded, we first plotted the time course of the Cantonese adults' target looking behavior during the test phase based on two analyses. In the first one, as originally planned when preparing the videos, the postnaming phase was aligned with the beginning of the onset consonant of the target words (see Figure 4, top panel). In the second analysis, we corrected the time course aligning the postnaming phase to the onset of the vowel (see Figure 4, bottom panel). On average, we corrected for 127 ms (range 32–235 ms). As can be seen from the comparison of the two figures, similar identification curves are found for the consonant and vowel conditions, with a very similar timing. While word recognition appears delayed in the tone condition compared to the other two conditions when recognition is time-locked to the onset of the consonant, this delay disappears when it is time-locked to the onset of the vowel. This suggests that tonal information is more likely available from vowel onset rather than consonant onset for the current set of pseudowords, and that the speed of use of tonal information in native processing is similar to that of consonantal and vocalic information.

Given the above findings, all analyses presented in the following sections are based on the recalculation of the pre/postnaming phase for the tone-contrasted trials, taking *vowel* onset +200 ms as the beginning of the postnaming phase. Note however that equivalent analyses time-locked to consonant onset provided the same pattern of results.

### Accuracy-overall analysis

**Figure 5:**
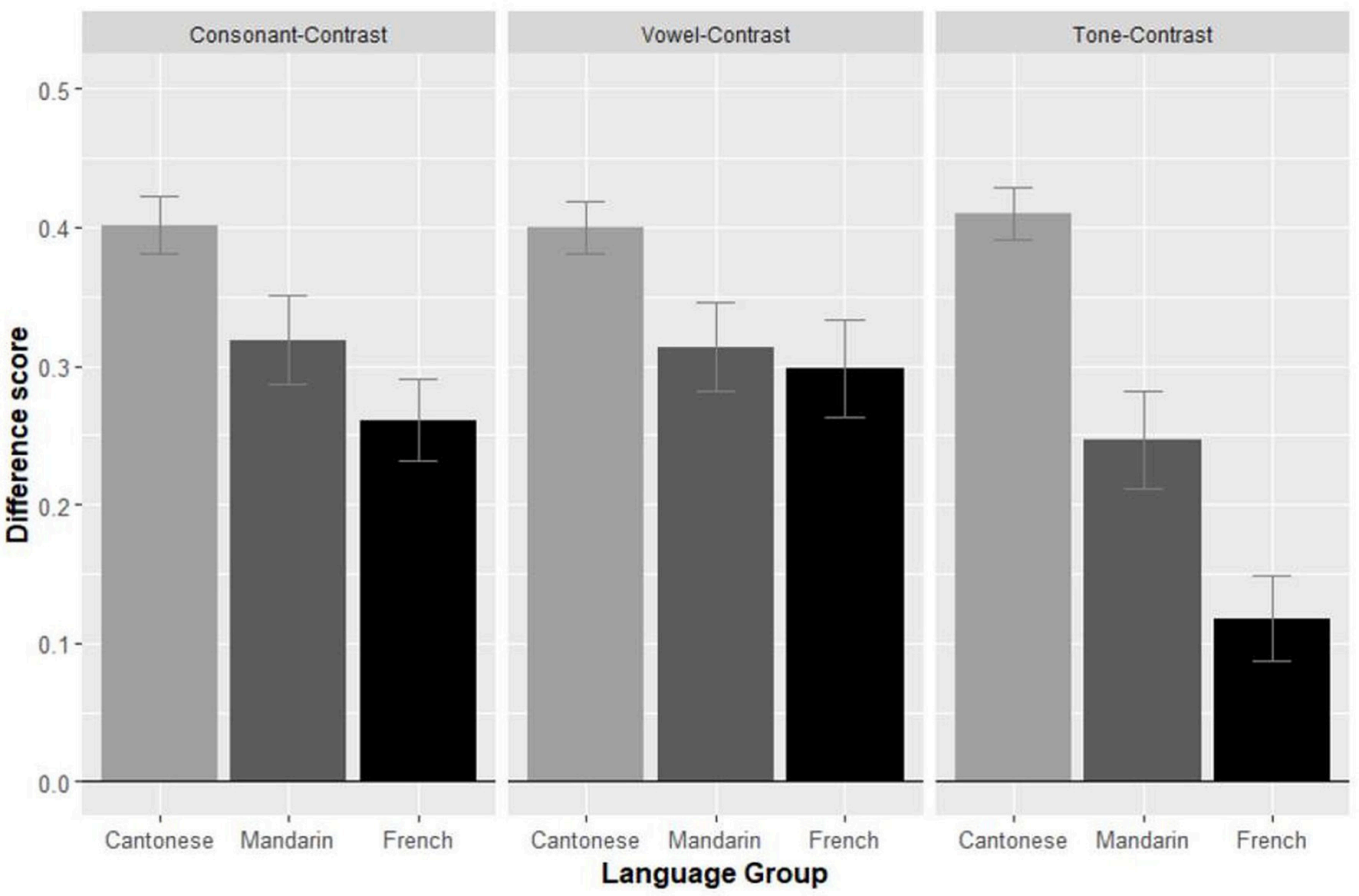
Size of the naming effect, broken down by the language of the participants (Cantonese, Mandarin and French) and the type of the contrast (consonant, vowel, tone). Error bars indicate standard errors of the means.

We first calculated the mean proportion of target looking (PTL = total looking time to target/ total looking time to both objects) on each trial for both the pre- and postnaming phase. For this purpose, two areas of interest (AOI) were defined (575 × 895 Pixel), each including one object. Time stamps that were not in any of the AOIs were treated as missing data, so that the calculated proportion of looking to one AOI is always relative to both AOIs, resulting in values between zero and 1 (i.e., a proportion value of 0.5 means that each AOI was looked at equally long). Word learning is typically reflected by the *naming effect* which corresponds to an increase in the proportion of target looking between the pre- and the post-naming phases that is significantly above 0 (e.g., Singh et al., 2015). The purpose of this prenaming correction is to control for looking preferences that are independent of the labeling. Difference scores between the pre- and postnaming phases were therefore calculated for each adult and each of the 24 contrast pairs, and then averaged for the 3 types of contrasts (see Figure 5). Zero corresponds to no increase in looking to target between the pre- and post-naming phases (chance performance). Positive difference scores mean an increase in target looking proportion.

For each of the three types of contrasts, adults in each language group exhibit an above chance naming effect (all *p*s < 0.001; see Table 2 for details). This establishes that adults in all language groups could learn the words in all conditions, even though all stimuli were in Cantonese, a language not known by the Mandarin- and French-speaking adults.

**Table 2:** Naming effect broken down by language and condition.

	**Mean (SD)**	**Comparison to 0 chance-level**
**CANTONESE-SPEAKING ADULTS:**		
Consonant trials	0.40 (0.104)	*t*_(23)_ = 18.93; *p* < 0.001
Vowel trials	0.40 (0.091)	*t*_(23)_ = 21.56; *p* < 0.001
Tone trials	0.41 (0.094)	*t*_(23)_ = 21.31; *p* < 0.001
**MANDARIN-SPEAKING ADULTS:**		
Consonant trials	0.32 (0.157)	*t*_(23)_ = 9.93; *p* < 0.001
Vowel trials	0.31 (0.156)	*t*_(23)_ = 9.87; *p* < 0.001
Tone trials	0.25 (0.171)	*t*_(23)_ = 7.05; *p* < 0.001
**FRENCH-SPEAKING ADULTS:**		
Consonant trials	0.26 (0.145)	*t*_(23)_ = 8.83; *p* < 0.001
Vowel trials	0.30 (0.172)	*t*_(23)_ = 8.46; *p* < 0.001
Tone trials	0.12 (0.150)	*t*_(23)_ = 3.83; *p* < 0.001

To test for differences between language group and type of contrast, a 2-way ANOVA with the main factors of native language (Cantonese, Mandarin, French) and type of contrast (consonant, vowel, tone) was performed. A main effect of language [*F*_(2, 69)_ = 16.96; *p* < 0.001] was found. *T*-tests revealed that Cantonese-speaking adults had a larger naming effect (0.40) than both Mandarin- [0.29, *t*_(46)_ = 3.92; *p* < 0.001] and French-speaking adults [0.22, *t*_(46)_ = 6.19; *p* < 0.001], whose performance did marginally differ [*t*_(46)_ = 1.92; *p* = 0.06]. This indicates an advantage of learning in one's native language vs. in an unknown language, and furthermore points toward a linguistic distance effect as Mandarin and Cantonese are related languages while French is unrelated to Cantonese.

There was also a main effect of type of contrast [*F*_(2, 138)_ = 10.46; *p* < 0.001], naming effects being larger for both consonants (0.33) and vowels (0.34) than for tones [0.26; *t*_(71)_ = 3.34, *p* = 0.001, and *t*_(71)_ = 3.34; *p* = 0.001, respectively]. This indicates that tone contrasts were overall more difficult to process than consonant and vowel contrasts. Performance between the consonant and vowel condition did not differ [*t*_(71)_ = 0.65, *p* = 0.51]. In addition, the native language x type of contrast interaction was significant [*F*_(4, 138)_ = 4.86; *p* = 0.001]. This indicates that performance for the different types of contrasts was differently affected in the three language groups. Compared to native Cantonese-speaking adults, both Mandarin- and French-speaking adults performed significantly worse on all three contrasts [tone: 0.25 vs. 0.41, *t*_(46)_ = 4.09; *p* < 0.001; 0.12 vs. 0.41, *t*_(46)_ = 8.06; *p* < 0.001; vowel: 0.31 vs. 0.40, *t*_(46)_ = 2.34; *p* = 0.02; 0.30 vs. 0.40, *t*_(46)_ = 2.57; *p* = 0.01; consonant: 0.32 vs. 0.40, *t*_(46)_ = 2.16; *p* = 0.04; 0.26 vs. 0.40, *t*_(46)_ = 3.86; *p* < 0.001]. Additionally, French-speaking adults performed worse on tone contrasts than Mandarin-speaking adults [0.12 vs. 0.25, *t*_(46)_ = 2.77; *p* = 0.008].

Comparing conditions within each language, taking all 8 trials per condition into account, no difference in performance between the consonant and vowel conditions was found for the three language groups [Cantonese speakers: 0.40 vs. 0.40, *t*_(23)_ = 0.07, *p* = 0.94; Mandarin speakers: 0.32 vs. 0.31, *t*_(23)_ = 0.24, *p* = 0.82; French speakers: 0.26 vs. 0.30, *t*_(23)_ = 1.16, *p* = 0.26]. Performance on tone contrasts was lower than in the other two conditions for French speakers [0.12 vs. 0.28, *t*_(23)_ = 5.14, *p* < 0.001], but not for Mandarin [0.25 vs. 0.32, *t*_(23)_ = 1.61, *p* = 0.12] and Cantonese speakers [0.41 vs. 0.40, *t*_(23)_ = 0.49, *p* = 0.63]. Redoing these analyses removing the Single Category trials and the Category Goodness trials in each condition (see details in Appendix) confirmed the lack of difference in performance between the 8 consonant and 5 vowel native-like/Two Category pairs for Mandarin [0.32 vs. 0.35, *t*_(23)_ = 0.93, *p* = 0.36], and the 6 consonant and 7 vowel native-like/Two Category pairs for French [0.29 vs. 0.32, *t*_(23)_ = 0.58, *p* = 0.56].

### Latency analysis

Second, following Havy et al. (2014), we examined the participants' latency in shifting from the distractor to the target object, that is the time needed to orient from the initially fixated distractor object to the target object after labeling. Faster latencies to the target object in a condition would indicate a processing advantage compared to the other conditions. In a first step, distractor-initial trials were defined as those in which participants fixated the distractor object at the onset of the pivotal phoneme (first consonant of the target word for consonant trials; first vowel for tone and vowel trials). These distractor-initial trials corresponded to, on average, 46% of all the trials (Cantonese: 45%; Mandarin: 45%; French: 48%). From those trials, we excluded trials in which participants shifted before the postnaming phase began (i.e., within the next 200 ms) as these saccades were probably programmed before the name of the target was processed (Cantonese: 21%; Mandarin: 22%; French: 10%) or did not shift at all (Cantonese: 1%; Mandarin: 6%; French: 7%) as well as outliers, that is values greater or smaller than 2.5 standard deviations from the mean (Cantonese: 1%; Mandarin: 2%; French: 2%).

Mean latencies and standard deviations are shown in Table 3 for each language and condition. We used a linear mixed model using the function *lmer* of the R package *lm4*, with random effects for participants and items (Bates et al., 2015), and the package *languageR* (Baayen, 2015) to obtain *p*-values. The model included fixed effects of condition (compared in sliding contrasts: Consonants-Vowels; Vowels-Tones), language group (also compared in sliding contrasts: French vs. Cantonese; Cantonese vs. Mandarin), and the interaction between condition and language group. We decided to test the consonant-vowel contrast to be able to compare with previously reported results, and the tone-vowel comparison because of the same target phoneme onset. As for the language contrasts, we took Cantonese as the native speaker reference group with which to compare both non-native speaker groups. The output measure was mean shift latency. The only significant differences were found between Cantonese and Mandarin participants (β = 163.31, *SE* = 50.23, *t* = 3.25, *p* = 0.002) and between Cantonese and French participants (β = −199.87, *SE* = 49.14, *t* = 4.07, *p* < 0.001), with the Cantonese participants having overall faster latencies then each of the two other language groups. This points, again, to a general native language advantage. Importantly, the conditions did not differ from each other or interact with language.

**Table 3:** Mean shift latencies in ms and their SDs (in brackets), broken down by language and condition.

**LANGUAGE**				
	**Cantonese**	**Mandarin**	**French**	**Mean (condition)**
**CONDITION**				
Consonants	369 (124)	489 (303)	548 (377)	469 (297)
Vowels	366 (260)	644 (514)	570 (386)	529 (408)
Tones	420 (281)	528 (325)	668 (395)	537 (350)
**Mean (language)**	386 (227)	543 (380)	592 (387)	

### Growth curve analysis

Third, we conducted a Growth Curve Analysis (GCA) which includes time as a predictor to estimate if differences between conditions emerged over time within each language group. As dependent measure we took the transformed proportion data during the postnaming phase using the empirical logit (*elog*, aggregated in 100 ms time bins) and analyzed it with a weighted mixed-effects linear regression model within the *eyetrackingR* package (modeled after Mirman et al., 2008). For each language group separately, we entered condition (again compared in sliding contrasts: Consonants-Vowels; Vowels-Tones), orthogonal polynomials (linear, quadratic and cubic time component), and the interaction between each time term and condition as fixed effects. Participants and items were entered as random effects into the model.

**Figure 6:**
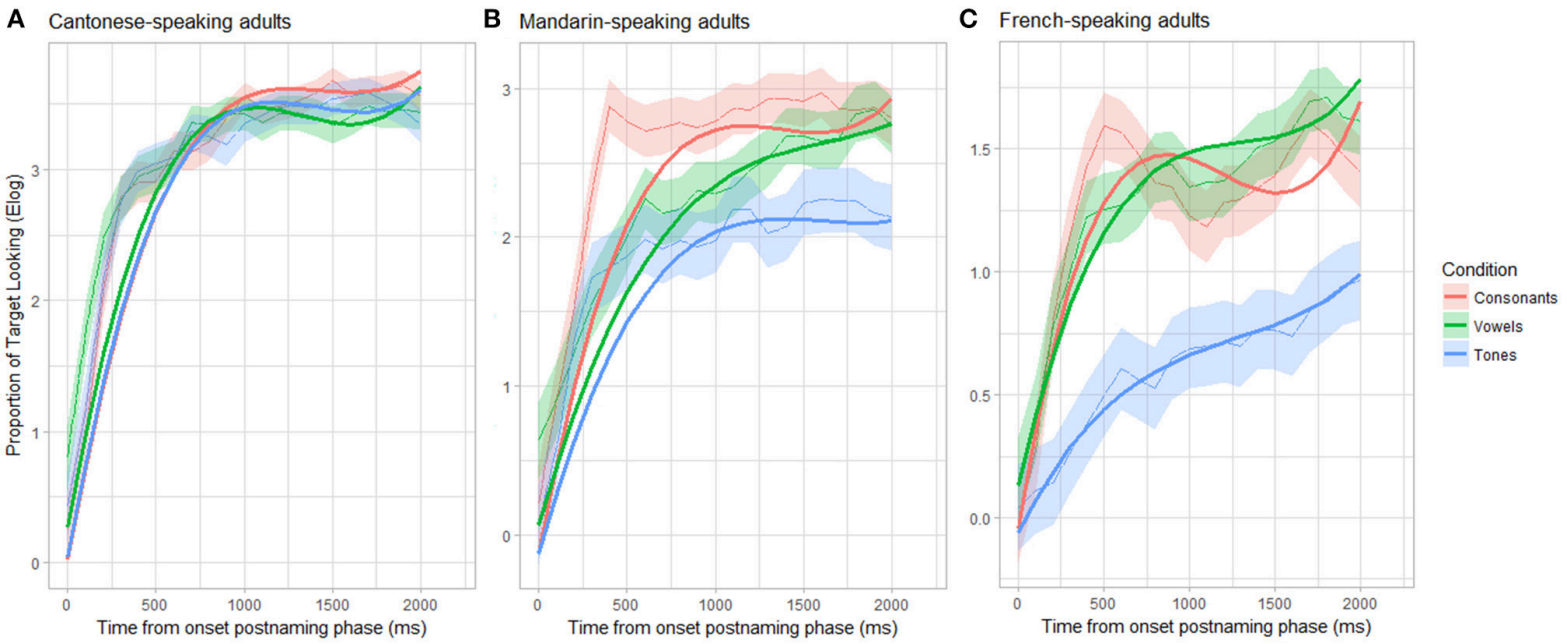
Time course during the postnaming phase of consonant, vowel and tone trials for Cantonese-**(A)**, Mandarin- **(B)**, and French-speaking adults **(C)**; shown as raw data (light) and fitted curves (bold).

For Cantonese-speaking adults (see Figure 6A), conditions (Consonant-Vowel; Vowel-Tone) did not differ in their mean target looking, but both contrasts interacted (marginally) significantly with time (linear parameter: β = −0.93, *SE* = 0.21, *p* < 0.001; β = 0.52, *SE* = 0.21, *p* = 0.01; quadratic parameter: β = 0.45, *SE* = 0.21, *p* < 0.03; β = −0.37, *SE* = 0.28, *p* = 0.08).

For Mandarin-speaking adults (see Figure 6B), there was no significant main effect of the Consonant-Vowel and the Vowel-Tone contrast (both *ps* > 0.22), indicating no differences in the overall target looking in the postnaming phase between those conditions. We found a significant interaction between the Consonant-Vowel contrast and time (specifically, the quadratic and cubic parameter: β = 0.94, *SE* = 0.28, *p* < 0.001; β = −0.72, *SE* = 0.28, *p* = 0.009), and between the Vowel-Tone contrast and time (linear parameter: β = −0.71, *SE* = 0.28, *p* = 0.01).

For French-speaking adults (see Figure 6C), the GCA revealed a significant main effect of the Vowel-Tone contrast on the intercept term, confirming the overall lower target fixations for the tone trials relative to the vowel trials (β = −0.70, *SE* = 0.19, *p* = 0.001). In addition, the Vowel-Tone contrast interacted (marginally) significantly with time (linear time parameter: β = −0.39, *SE* = 0.21, *p* = 0.06; quadratic time parameter: β = 0.43, *SE* = 0.21, *p* = 0.04), suggesting divergent linear and non-linear temporal trajectories for tone and vowel trials. While the Consonant-Vowel contrast on the intercept term was not significant (β = 0.05, *SE* = 0.19, *p* = 0.81), its interaction with time was marginally significant (linear time parameter: β = 0.38, *SE* = 0.21, *p* = 0.06; cubic time parameter: β = −0.40, *SE* = 0.21, *p* = 0.06). This indicates that the temporal trajectory tends to differ between these conditions, although these differences are only trends, in line with the lack of mean target looking time differences during the postnaming phase between the consonant and vowel trials.

Note that *eyetrackingR* fits curves using orthogonal polynomials so that the estimated time parameters are independent from each other. As a consequence, the condition effect on the intercept corresponds to differences averaged across the entire postnaming phase. In a second model, we used natural polynomials in order to obtain so-called anticipatory effects, that is mean differences between conditions at the onset of postnaming phase (see Barr, 2008). These analyses revealed no significant effect of condition on the intercept term (all *p*s > 0.35). Thus, it can be ruled out that differences between conditions were already present before the postnaming phase started, that is before the critical information in a trial was processed.

### Cluster-based permutation analysis

To further explore the different temporal trajectories that the results of the CGAs indicated, we conducted a cluster-based permutation analysis (Maris and Oostenveld, 2007) for each language group separately to identify the exact time periods where conditions differ significantly from each other. As dependent measure we took the proportion of target looking within each 100 ms bin across the postnaming phase (20 bins). In a first step, this analysis compares conditions at each time bin with a *t*-test and identifies any time period(s) of adjacent bins in which conditions significantly differ. As *t*-threshold we chose an α-level of 0.05 (two-tailed). This yields in cluster-level *t*-value(s) which correspond to the sum of all single sample *t*-values within the time period(s). In a second step, it generates a Monte-Carlo distribution to compare the cluster-level *t*-value(s) by randomly assigning the trials to conditions and repeating step 1 several times (for our data: 1000 times). This results in a Monte Carlo *p*-value for each observed time cluster which reflects the probability that this cluster could have occurred simply by chance.

This analysis revealed no differences between conditions for the Cantonese-speaking group. For Mandarin-speaking adults, Consonant and Vowel trials diverged from 300 to 900 ms during the postnaming phase (cluster *t* = 16.29, Monte Carlo *p* = 0.02) with Consonant trials having higher target looking proportions. While Tone trials did not differ from Vowel trials, they did from Consonant trials between 400 and 2000 ms during the postnaming phase (cluster *t* = 51.91, Monte Carlo *p* < 0.001), again Consonant trials having higher target looking proportions. Interestingly, redoing these analyses removing the Single Category and Category Goodness trials in each condition (see details in Appendix) there was no difference between conditions any more. For French-speaking adults, two significant clusters were found: both Tone and Vowel trials and Tone and Consonant trials diverged from 200 to 2000 ms during the postnaming phase (cluster *t* = 63.43, Monte Carlo *p* < 0.001; cluster *t* = 62.27, Monte Carlo *p* < 0.001, respectively), with Tone trials having lower target fixations. Consonant and Vowel trials did not differ. This was still the case after removing the Single Category and Category Goodness trials in the vowel and consonant conditions (see details in Appendix).

## Discussion

In this study, we investigated whether and how adults can quickly learn new minimal pair words in a non-native tone language, Cantonese, and whether this ability is modulated by native phonological knowledge. We tested this learning ability in Mandarin- and French-speaking adults, using Cantonese pseudowords differing minimally in either a consonant, a vowel, or a tone, and compared their performance to those of native Cantonese-speaking adults. Overall, we found that all three groups of adults performed at above chance levels in learning the pseudowords, and this held for all three types of contrasts. Also, compared to native Cantonese-speaking adults, both Mandarin- and French-speaking adults performed worse on all three types of contrasts. Furthermore, French-speaking adults performed even worse on tones when compared to Mandarin-speaking adults.

The present findings first establish that adults in all three language groups could rapidly learn new words in a computer-based situation, after solely 6 repetitions of each word. Note that the present interpretation in terms of word-learning needs to be qualified by the fact that the present study does not establish long-term establishment of lexical items, and could result from simple associations between the pseudowords and either the objects (or the side of the screen on which the objects were presented). Future studies will have to further probe our word-learning interpretation, using designs testing for word learning independent of object localization, and in long term memory, for example adapting the word-learning design used in Dittinger et al. (2016). While this word-learning finding is in part trivial for the Cantonese-speaking adults (though see more discussion on this below), it holds even when the new words were presented to Mandarin- and French-speaking adults, for whom Cantonese was a non-native language, and who had no knowledge of Cantonese prior to taking part in the experiment. Our findings reveal a significant effect of nativeness status, as overall, Cantonese-speaking adults performed better than the other two groups (in overall performance and shift latency analyses).

The effect of linguistic distance is less clearcut. Indeed, although Cantonese is closer to Mandarin than to French (at many levels including phonology, morphology and syntax, Li, 1937; Gong, 1980; DeLancey, 2009), this did not significantly impact overall performance and shift latencies, as French-speaking adults performed at the same overall level as Mandarin-speaking adults, in spite of a trend in the expected direction for overall performance (see further discussion in the Appendix for a more fine-grained approach). Our findings thus establish robust word learning abilities in a non-native language in adulthood, that contrast with the difficulties that adults have in learning some specific aspects of the phonology and syntax of non-native languages (e.g., Flege et al., 1999; Birdsong and Molis, 2001; Dupoux et al., 2008; Boll-Avetisyan et al., 2016). This difference might be due to the fact that while the acquisition of the phonology and syntax of one's native language is to a great extent completed in the first years of life, vocabulary acquisition is a lifelong, continuing process that allows for the acquisition of specialized vocabularies (as when, for example, becoming a -developmental- psychologist!) or learning the names of new objects and concepts (e.g., to “log into” a “googledoc” on one's “iphone”) in the native language.

Importantly, these word learning abilities were found in a specific learning context in which adults had to learn words presented in pairs, and in which the sound forms of the two words differed only by a consonant, vowel or tone. The fact that Mandarin- and French-speaking adults succeeded in learning the word pairs in all three conditions establishes that they could process fine segmental (consonantal and vocalic) and suprasegmental (tonal) information in doing so, and that they were establishing representations of the word forms that included specific segmental or tonal information. This finding is particularly striking for the French speakers' performance with tone contrasts, given that tones are not used in French at the lexical level. It could be due to the fact that these contrasts were introduced to the adults in minimal pairs of words, where they had to pay attention to the fine phonetic detail in order to distinguish the objects and memorize the words. Further research will be needed to explore whether our French-speaking adults would have failed to use such precise phonetic information if they had not been presented with minimal pairs, leading to lower or at chance performance. Importantly though, the ability of the French-speaking adults to use tonal information when learning words suggest that the rebound in tone discrimination found in late infancy in Dutch, another non-tonal language (Liu and Kager, 2014), interpreted in relation to the acquisition of the intonation of the native language, would not be limited to low levels of processing, but would extend to the lexical level.

Our findings also establish that adult performance is not solely based on the acoustic distance between the contrasted sounds, but is also dependent on their native phonological system. At this more fine-grained level, language distance appears to play a role, as our results clearly show that the Mandarin-speaking adults performed better than the French-speaking adults in learning words distinguished by Cantonese tonal contrasts. Since there was no difference in performance between the two language groups for consonants and vowels, this effect likely indicates that Mandarin-speaking adults, as experienced tone language users, exhibit greater ability in processing non-native tonal information at the lexical level, when compared to the non-tone user French speakers. In the Appendix, we present exploratory analyses, based on individual trials analyses, that allow some evaluation of the Perceptual Assimilation Model (PAM; for consonants: Best, 1995; for vowels: Tyler et al., 2014; for tones: Hallé et al., 2004) applied here at the level of word learning rather than speech processing.

Besides providing data on the phonological/lexical interface in processing a new, non-native language, our results also provide an evaluation of the use of tonal information in word learning, and its impact on processing consonantal and vocalic information at the lexical level in native speakers of a tone language. Regarding the use of tonal contrasts, we found that tonal contrasts are as important as consonantal and vocalic contrasts in processing word meanings for native Cantonese-speaking adults. This is revealed by the overall accuracy analyses showing that Cantonese adults perform at the same level in all three contrast conditions. Our time course analysis further shows that all three kinds of contrasts are processed at the same speed from the onset of the contrasting phonemes. For the tones, the comparison of our two analyses time-locked to consonant vs. vowel onset suggests that tonal information became available from the onset of the vowel. This might be related to the fact that 6 of the 8 pairs we presented started with unvoiced consonants, so that tonal information was mostly carried by the vowels. Whether a similar pattern would be found for syllables starting with voiced consonants would need to be evaluated in an experimental design counterbalancing the two types of consonants.

Furthermore, our work bears on the issue of the relative weight given to consonantal and vocalic information in lexical processing. Previous studies on various Indo-European languages (English, Dutch, French, Italian, Spanish) have found that adults have a consonant bias in accessing or learning words (e.g., van Ooijen, 1996; Cutler et al., 2000; Bonatti et al., 2005; Creel et al., 2006; New et al., 2008; Toro et al., 2008; Carreiras et al., 2009; Delle Luche et al., 2014; Havy et al., 2014; New and Nazzi, 2014). This supports the “division of labor” proposal by Nespor et al. (2003) that consonants are given more weight than vowels in lexical processing (while vowels are given more weight than consonants at the prosodic/syntactic levels). Accordingly, in the present study, we investigated Cantonese, a tone language, where lexical meanings are also crucially cued by tones. Our interest came from the fact that since tones are essentially associated with the voiced portions of syllables, which mostly correspond to vowels (and nasal codas) in Cantonese, and since only a few onset consonants (/j, w, m, n, η/) are voiced in that language, the relative weight given to consonants and vowels might be different from what has been found for Indo-European languages. The effect of tones could either increase the weight given to vowels (since they carry both segmental and tonal information, compared to only segmental information in non-tone languages) or decrease their weight even further (due to additional acoustic variation related to tonal differences and the fact that each vowel in Cantonese can carry 6 different tones).

Our findings did not reveal any advantage for either consonantal or vocalic information in lexical processing for Cantonese-speaking adults, as shown by their overall similar performance in the consonant and vowel contrast conditions. This finding differs from all previous findings on adult speakers of non-tonal languages, and in particular with the findings of a clear C-bias in latency analyses found when French adults learn new words (Havy et al., 2014). The present null effect (lack of difference between the C and V conditions), which thus needs to be interpreted with caution, might be taken as evidence that Cantonese-speaking adults pay more attention to vowels that carry tonal information than non-tone language users, resulting in a lack of C-bias. This interpretation needs to be considered cautiously given that a null effect was also found in the other two language groups, including in the French-speaking adults, which have been documented to have a C-bias in lexical processing when processing words in their native language (e.g., Bonatti et al., 2005; New et al., 2008; Havy et al., 2014). This lack of effect in the French- and Mandarin-speaking adults could mean that there is something in the acoustics of the stimuli used in the present study that does not support a C-bias. Alternatively, it could mean that the C-bias only operates in the native language, or in languages in which adults have sufficient experience/knowledge, hence the null effect found here for the French-speaking (and Mandarin-speaking) adults who had no or limited knowledge of Cantonese. Importantly though, our interpretation in terms of lack of a C-bias in Cantonese is corroborated by two recent studies having explored similar issues in either Mandarin- (Wiener and Turnbull, 2016) or Cantonese-speaking (Gómez et al., 2017) adults, using a word reconstruction and word form segmentation task respectively. As discussed in the introduction, their findings differ from those previously found in Indo-European languages, failing to find a clear C-bias in both languages, thus suggesting a different balance in the weight given to consonants and vowels in these two tone languages.

The above findings that begin to establish crosslinguistic differences in consonant/vowel weight between adult listeners of tonal vs. non-tonal languages are to be considered in relation to infant studies on Indo-European languages that have shown that the C-bias is modulated in infancy both developmentally and crosslinguistically (see Nazzi et al., 2016, for a complete review). Indeed, results from French and Italian show that while a C-bias is found from 8 months onward (Nazzi, 2005; Hochmann et al., 2011; Poltrock and Nazzi, 2015; Nishibayashi and Nazzi, 2016), it is not present up to 6 months of age (Benavides-Varela et al., 2012; Bouchon et al., 2015; Nishibayashi and Nazzi, 2016; Hochmann et al., 2018). Moreover, a C-bias could not be attested before 30 months in British English-learning infants (Nazzi et al., 2009; Floccia et al., 2014), and Danish-learning 20-month-olds demonstrate a V-bias (Højen and Nazzi, 2016). Taken together, these studies suggest that the C-bias is acquired and that its acquisition depends on the phonological and lexical properties of the native language.

Given that Cantonese- and Mandarin-speaking adults appear to have a reduced or reversed bias (Wiener and Turnbull, 2016; Gómez et al., 2017; present study), it is of great interest to expand research on the consonant bias to infants and toddlers learning a tone language, which was one of the original motivations for setting up the present study. At present, only one study has started to explore this issue in (Mandarin-dominant) Mandarin-English bilingual toddlers (aged 2.5–3.5 years) and preschoolers (aged 4–5 years). In a word recognition task exploring their sensitivity to mispronunciations of known words, the toddlers were found to be more sensitive to tone than consonant and vowel mispronunciations, while the reverse pattern was found in preschoolers (Singh et al., 2015). However, at both ages, no differences in sensitivity were found between consonant and vowel mispronunciations. Future studies will have to expand on this first finding, exploring such effects in younger monolingual infants learning various tone languages, and exploring various aspects of lexical processing, including both word learning and lexical comprehension.

In conclusion, the present study establishes adults' word learning abilities in an unknown language, and show that level of performance is modulated by how the phonologies of the native and non-native languages map onto each other. They also bring evidence suggesting that being a speaker of a tonal language reduces the consonant bias in lexical processing previously found in adults of several Indo-European languages, probably due to the fact that tones are carried by vowels more than by consonants. However, no clear bias could be found for either consonants or vowels, and future studies will have to further probe the link between phonological and lexical processing in tone languages. These findings nevertheless set up the foundations for equivalent developmental studies that will inform our understanding of what determines the phonological biases that are observed in lexical processing.

## Author contributions

SP created the experimental stimuli and design, conducted data analyses and drafted the manuscript. CK assisted in stimuli creation, recruited participants and collected data in Hong Kong. HuiC interpretated the data and drafted the manuscript. HC and TN conceptualized the study and supervised all stages of the project.

### Conflict of interest statement

The authors declare that the research was conducted in the absence of any commercial or financial relationships that could be construed as a potential conflict of interest.
