# Supplementary material for: Adult Learning of Novel Words in a Non-native Language: Consonants, Vowels, and Tones
Source: Front Psychol. 2018 Jul 24;9:1211. doi: 10.3389/fpsyg.2018.01211 (PMC6066720; doi:10.3389/fpsyg.2018.01211)
Supplement: Supplementary file 1 [file Data_Sheet_1.docx]

**APPENDIX**

Our study also provides preliminary findings that address the issue of whether predictions based on the Perceptual Assimilation Model (PAM; Best, 1995; Best & Tyler, 2007) could explain differences in performance between individual trials in a word learning task. The PAM was proposed to account for why decrease in discrimination/identification performance is not found for all non-native sounds, as had been revealed by the fact that English-speaking infants and adults maintain sensitivity to some Zulu click contrasts (Best et al., 1988). It was later extended to vowel processing (Tyler et al., 2014) and the perception of suprasegmentals (Hallé et al., 2004; So, 2010; So & Best, 2008, 2010, 2014).

Overall, the PAM states that performance in low-level identification/discrimination of non-native sounds will depend on whether, and if so how, these sounds assimilate to the sound categories of the native language. Hence, performance should be excellent if the two non-native sounds assimilate to two different native phonological categories (‘Two Category’), while it should be particularly affected if the two non-native sounds assimilate equally well or poorly to a single native category (‘Single Category’). However, performance could vary from moderate to very good (depending on acoustic distance) if one non-native sound assimilates to a native category better than the other (‘Category Goodness’). Also, if the two non-native sounds do not assimilate to any native category, performance would depend on how acoustically distinct the two sounds are. Note that recent PAM accounts for suprasegmentals propose that a non-native prosodic category will either primarily assimilate to some native category (*categorized* prosodic category, corresponding to a situation in which a specific non-native tone might assimilate to a native tone, but also to a native intonational pattern or pitch accent, as assimilations can cross different suprasegmental types), or not (*uncategorized* prosodic category; see So & Best, 2014). Accordingly, it was found that Mandarin Tone 1 /55/ is categorized as an exemplar of a native prosodic category by adults from different language groups (either as T1 /55/ in Cantonese, high-high (HH) pitch accent in Japanese, or *Statement* intonation in English), but it is uncategorized and perceived with its core phonetic features that fall in between two native prosodic categories (i.e., *Statement* and *Exclamation*) by French adults (So, 2010; So & Best, 2008, 2010, 2014). Would similar PAM effects carry to a higher processing level, namely word learning?

In the present study, most consonant and vowel contrasts had been chosen so that the contrasted sounds would assimilate to different native categories for both our Mandarin- and French-speaking participants, and most tone contrasts had been chosen such that the contrasted tones would assimilate to different Mandarin tones. This choice was motivated by our initial goal, which was to establish the possibility of using phonetic/phonological information at the lexical level when learning words in a non-native language. However, because phonologies do not map neatly across languages (in particular when conducting a crosslinguistic study involving three different languages), a few of the contrasts that we used turned out not to be phonologically contrastive in French and/or Mandarin. Therefore, we conducted exploratory analyses of accuracy for individual trials (see Fig. 7) to determine whether non-native performance (in French- and Mandarin-speaking adults) was modulated according to whether the contrast in a given trial was likely to correspond to Two Category (which should be easy to discriminate), Single Category (which should be hard to discriminate) or Category Goodness (level of discrimination dependent on acoustic distance) assimilations in their native language, as predicted by PAM. For the tone contrasts, we did not make predictions according to how different tones are categorized into the native prosodic categories in Mandarin and in French, as we did not carry out a categorization task as previous studies did (So, 2010; So & Best, 2008, 2014). Rather, we based our predictions on phonological and phonetic terms (So & Best, 2010).

*Consonant contrasts*

According to PAM, French speakers should have difficulties in discriminating the two Cantonese consonants /k^h^/ and /k/ and as they should assimilate equally well to the French phoneme /k/ (which has aspirated and unaspirated allophones in actual speech, even though aspiration is not phonemic in French) and form a Single Category; the same should hold for /ts/ and /ts^h^/. As can be seen in Figure 7a, French-speaking adults performed significantly worse on those 2 contrasts (0.15) compared to the remaining 6 native-like contrasts (0.29), *t*(23) = 2.67, *p* = .01. For Mandarin speakers, all these consonants can be considered as native-like (given the possibility that nonnative /kw^h^/ might assimilate to the native phoneme sequence of /k^h^u/, a stop plus a glide, in Mandarin), which would constitute Two Category pairs according to PAM. As predicted, the performance on all these consonant contrasts remained high in the Mandarin-speaking adults (mean: 0.32, range: 0.28-0.38).

*Vowel contrasts*

For vowels, according to PAM, confusions would likely be made with the pair /m**ɐ**u.t^h^u/-/m**a**u.t^h^u/ for both Mandarin and French speakers, and with the pairs /h**œ**2.t^h^i1/ - /h**ɔ**2.t^h^i1/ and /k**ɛ**1.tsɛ1/ - /k**œ**1.tsɛ1/ for Mandarin speakers, as they involve Category Goodness assimilations. The /**a**u/ - /**ɐ**u/ pair involves a length distinction that is not contrastive in these two languages, where /au/ would typically assimilate to the diphthong /au/ (Mandarin) or the vowel sequence /a.u/ (French; such as in ‘c**aou**tchouc’) while /ɐu/ would likely do the same though less typically. The /œ/ - /ɔ/ and /ɛ/ - /œ/ pairs involve a front-mid-rounded vowel /œ/ that does not exist in Mandarin, and would possibly assimilate to either /ɔ/ or /ɛ/. As can be seen in Figure 7b, low performance for the /m**ɐ**u.t^h^u/-/m**a**u.t^h^u/ pair is confirmed in Mandarin, as performance on this contrast was significantly lower than the average naming effect of the 5 native-like vowel contrasts (M = 0.12 versus 0.37, *t*(22) = 2.45, *p* = .02). In French-speaking adults, however, this effect was only a trend as the performance for the /mɐu.t^h^u/-/mau.t^h^u/ pair was not significantly different from the 7 other native-like vowel contrasts in French (French: 0.17 versus 0.32, *t*(23) = 1.68, *p* = .11). In addition, in Mandarin, performance on the 2 pairs involving the non-native vowel /œ/ (/h**œ**2.t^h^i1/- /h**ɔ**2.t^h^i1/ and /k**ɛ**1.tsɛ1/ - /k**œ**1.tsɛ1/) was not significantly different from the average naming effect of the 5 other native-like vowel contrasts in Mandarin (M = 0.34 versus 0.35, *t*(23) =-0.23, *p* = .82). This indicates that Mandarin adults might be able to represent the nonnative vowel /œ/ in the task, which is likely due to the fact that the vowel features *place* and *roundness* are both used to distinguish vowels in Mandarin.

*Tone contrasts*

As mentioned earlier, given our lack of categorization data of the Cantonese tones by Mandarin and French speakers, we only discuss here predictions for Mandarin adults based on phonological and phonetic similarities between Mandarin and Cantonese tones. Of the 8 tone pairs, the T1-T2 pair was considered to correspond to Two Category assimilation in Mandarin, as both Cantonese and Mandarin distinguish a high level T1 /55/ tone and a high rising T2 /25/ or /35/ tone (Cantonese: Bauer & Benedict, 1997; Hashimoto, 1972; Yip, 2002; Mandarin: Cheng, 1966; Duanmu, 2000; Howie, 1976; Wang, 1963; see also Figure 1). In contrast, the T2-T6, T1-T3 (used in 2 pairs) and T1-T4 pairs all involved a native-like tone in Mandarin (either the high level tone T1 /55/ or the high rising tone T2 /25/), and a non-native tone (either the Cantonese T6 /22/, T3 /33/, or T4 /21/). According to PAM, Mandarin speakers might or might not have difficulty in discriminating these pairs as they might constitute Category Goodness assimilations, depending on how phonetically (dis)similar they are. However, as these pairs all differed in a similar pitch feature, i.e., high versus non-high, which is considered to be perceptually salient (Gandour, 1978; Harrison, 1998, 1999, 2000), performance was predicted to be good. As shown in Figure 7c, performance on the corresponding 4 trials patterned as the native-like contrast T1-T2 in Mandarin (0.35 vs. 0.39, *t*(22) = 0.44; *p* = .67), therefore we grouped these 5 pairs together in our subsequent analyses.

On the other hand, the pairs T5-T6, T3-T4 and T4-T6 did not involve any native-like tones in Mandarin. As predicted by PAM, they all should be difficult to discriminate by Mandarin speakers, as either the two non-native tones should assimilate equally poorly to the same native category (forming a Single Category) or they should both be unassimilable to any categories while sharing similar pitch properties. As can be seen on Figure 7c, those were the contrasts for which performance (mean naming effect: 0.07) was at chance level (*t*(23) = 0.93, *p* = .18) and significantly worse than performance for the other 5 pairs (0.35, *t*(23) = 3.30, *p* = .003). In line with PAM, Mandarin speakers’ performance on these 3 non-native contrasts (0.07) was closer to that of the French speakers (0.12, *t*(46) = 0.57, *p* = .57) than that of the Cantonese speakers (0.34, *t*(46) = 3.30, *p* < .001), while Mandarin speakers’ performance on the other 5 contrasts (0.35) was closer to that of the Cantonese speakers (0.44, *t*(46) = 1.92, *p* = .06) than that of the French speakers (0.12, *t*(46) = 3.77, *p* < .001).

These individual trial results provide some evaluation of the Perceptual Assimilation Model (PAM; for consonants: Best, 1995; for vowels: Tyler et al., 2014; for tones: Hallé et al., 2004; So, 2010; So & Best, 2008, 2010, 2014) applied here at the level of new word learning rather than that of speech processing. PAM predicts that performance on the few trials involving non-native sounds or contrasts that PAM would be lower than performance on the more numerous trials that involve contrasts that should assimilate to native contrasts. This was the case for the /k/ - /k^h^/ and /ts/ - /ts^h^/ pairs for French speakers, and the T5-T6, T3-T4 and T4-T6 pairs for Mandarin speakers. Performance was more mixed for Category Goodness pairs, for which PAM would predict variable levels of performance, probably depending on the acoustic distance between the two sounds. We found that performance was low on the /au/ - /ɐu/ pair for both French and Mandarin speakers (though not significantly different from the other vowel contrasts in French speakers), but it was high on the /œ/ - /ɔ/ and /ɛ/ - /œ/, and T2-T6, T1-T3 and T1-T4 pairs for Mandarin speakers. Taken together, these findings provide some evaluation of PAM beyond speech processing, suggesting that the effects explained by PAM at that level might extend (at least in part) to the processing of phonetic information at the lexical level. However, because the goal of the present study was to establish word learning in a non-native language, the status of the pairs in terms of PAM predictions was not counterbalanced (in fact, we had chosen as many native-like contrasts as possible) so that more careful exploration of this issue will require further experimentation.


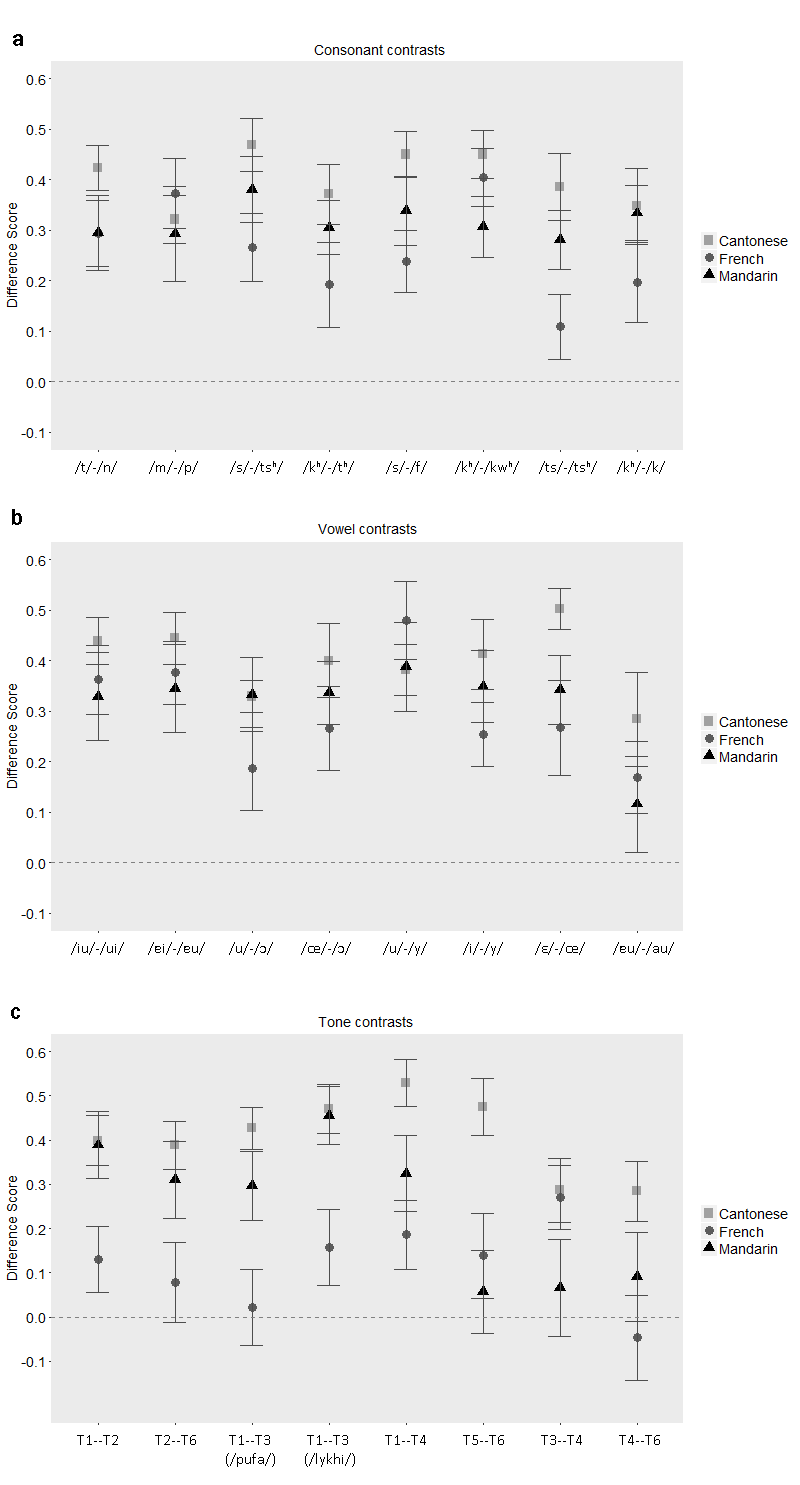


*Figure 7*. Size of naming effect, broken down by the language of the participants (Cantonese, Mandarin and French) and the individual contrast (separated for consonant (a), vowel (b) and tone (c) trials). Error bars indicate standard errors of the means.

**REFERENCES**

Best, C.T. (1995). A direct realist view of cross-language speech perception. In Strange, W.

(Ed.), *Speech Perception and Linguistic Experience: Issues in Cross-Language Research*.

New York, 171-204.

Best, C.T., McRoberts, G.W. & Sithole, N.M. (1988). Examination of perceptual reorganization

for nonnative speech contrasts: Zulu click discrimination by English-speaking adults and

infants. *Journal of Experimental Psychology: Human Perception and Performance, 14*, 345-

360.

Best, C.T., & Tyler, M. (2007). Nonnative and second-language speech learning: The role of

language experience in speech perception and production. In O.D.Bohn and M. Munro (Eds),

*Language Experience in Second Language Speech Learning: In Honor of James E. Flege*.

John Benjamins: Amsterdam, 13-24.

Gandour, J. (1978). The perception of tone. In A.V. Fromkin (Ed.). *Tone: A Linguistic Survey*

(pp. 41-76), New York: Academic Press.

Hallé, P.A., Chang, Y.-C., & Best, C.T. (2004). Identification and discrimination of Mandarin

Chinese tones by Mandarin Chinese vs. French listeners. *Journal of Phonetics* 32*,* 395–421.

Harrison, P.A. (1998). Yoruba babies and unchained melody. *UCL Working Papers in Phonetics,*

*10*: 33-52.

Harrison, P.A. (1999). *The Acquisition of Phonology in the First Year of Life*. PhD Dissertation,

University College London.

Harrison, P.A. (2000). Acquiring the phonology of lexical tone in infancy. *Lingua, 110*: 581-616.

So, C.K. (2010). Categorizing Mandarin tones into Japanese pitch-accent categories:

The role of phonetic properties. In *Proceedings of Interspeech 2010 Satellite Workshop*

*on Second Language Studies*, *Tokyo*.

So, C.K., & Best, C.T. (2008). Do English speakers assimilate Mandarin tones to English

prosodic categories? In *Proceedings of Interspeech 2008* (p. 1120). Baixas, France.

So, C.K., & Best, C.T. (2010). Cross-language perception of non-native tonal contrasts: Effects

of native phonological and phonetic influences. *Language & Speech,* *53*, 273–293.

So, C.K., & Best, C.T. (2014). Phonetic influences on English and French listeners’ assimilation

of Mandarin tones to native prosodic categories. *Studies in Second Language Acquisition, 36*,

195-221. doi:10.1017/S0272263114000047
